# 4-Hydr­oxy-2,2,6,6-tetra­methyl­piperidinium perchlorate

**DOI:** 10.1107/S1600536808002997

**Published:** 2008-03-05

**Authors:** Ying Cui, Yun-Hui Zhang, Peng-Wei Zhang

**Affiliations:** a School of Pharmaceutical Science and Technology, Tianjin University, Tianjin 300072, People’s Republic of China

## Abstract

In the title salt, C_9_H_20_NO^+^·ClO_4_^−^, inter­molecular hydrogen bonds are observed, which determine the crystal packing.

## Related literature

For general background, see Borzatta & Carrozza (1991 ▶). 
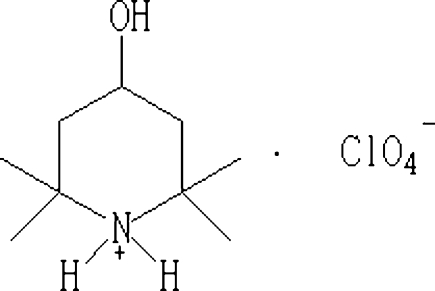


## Experimental

### 

#### Crystal data

C_9_H_20_NO^+^·ClO_4_^−^
*M* *_r_* = 257.71
Monoclinic, 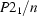

*a* = 7.5712 (15) Å
*b* = 13.927 (3) Å
*c* = 12.007 (2) Å
β = 100.71 (3)°
*V* = 1244.0 (4) Å^3^
*Z* = 4
Mo *K*α radiation
μ = 0.31 mm^−1^
*T* = 113 (2) K
0.12 × 0.04 × 0.04 mm

#### Data collection

Rigaku Saturn diffractometer
Absorption correction: multi-scan (*CrystalClear*; Rigaku/MSC, 2005 ▶) *T*_min_ = 0.963, *T*_max_ = 0.988
7480 measured reflections
2183 independent reflections
1797 reflections with *I* > 2σ(*I*)
*R*_int_ = 0.046

#### Refinement

*R*[*F* ^2^ > 2σ(*F* ^2^)] = 0.045
*wR*(*F* ^2^) = 0.127
*S* = 1.10
2183 reflections
157 parameters
H atoms treated by a mixture of independent and constrained refinement
Δρ_max_ = 0.60 e Å^−3^
Δρ_min_ = −0.48 e Å^−3^

### 

Data collection: *CrystalClear* (Rigaku/MSC, 2005 ▶); cell refinement: *CrystalClear*; data reduction: *CrystalClear*; program(s) used to solve structure: *SHELXS97* (Sheldrick, 2008 ▶); program(s) used to refine structure: *SHELXL97* (Sheldrick, 2008 ▶); molecular graphics: *SHELXTL* (Sheldrick, 2008 ▶); software used to prepare material for publication: *SHELXTL*.

## Supplementary Material

Crystal structure: contains datablocks I, global. DOI: 10.1107/S1600536808002997/hg2373sup1.cif

Structure factors: contains datablocks I. DOI: 10.1107/S1600536808002997/hg2373Isup2.hkl

Additional supplementary materials:  crystallographic information; 3D view; checkCIF report

## Figures and Tables

**Table 1:** Hydrogen-bond geometry (Å, °)

*D*—H⋯*A*	*D*—H	H⋯*A*	*D*⋯*A*	*D*—H⋯*A*
N1—H1*A*⋯O4^i^	0.92 (3)	2.05 (3)	2.914 (3)	157 (2)
N1—H1*B*⋯O1^ii^	0.88 (3)	1.97 (3)	2.847 (3)	173 (2)
O1—H1⋯O2^iii^	0.82	2.09	2.896 (2)	167
O1—H1⋯Cl1^iii^	0.82	2.93	3.6985 (16)	158

Symmetry codes: (i) 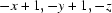; (ii) 
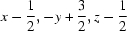
; (iii) 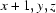.

**Fig. 1.**
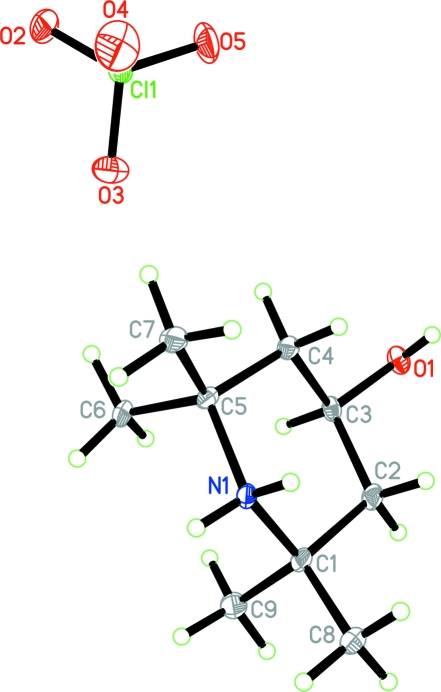
A view of the molecule (I). Displacement ellipsoids are drawn at the 30% probability level and H atoms are shown as small spheres of arbitrary radii.

## supplementary crystallographic information

### Comment

2,2,6,6-Tetramethyl-4-hydroxy-piperidin-4-ol is a very important intermediate in the synthesis of hindered light stabilizers (Borzatta & Carrozza, 1991). We report here the crystal structure (2,2,6,6-tetramethyl-4-hydroxypiperidinium perchlorate) (Fig. 1).

Intermolecular N—H···O, O—H···O, O—H···Cl hydrogen bonds are observed which help to establish the crystal packing. The piperidine ring adopts chair conformation.

### Experimental

2,2,6,6-tetramethylpiperidin-4-ol (3.2 mmol,0.5 g) was dissolved in perchloric acid solution(2.5 mol/l, 3 ml). Block shaped colorless crystals grew with slow evaporation of solvent.

### Refinement

All H atoms were constrained; positioned geometrically (C—H = 0.99–1.00 Å) and refined as riding with *U*_iso_(H) = 1.2*U*_eq_(carrier) or 1.5_eq_(methyl groups).

### Crystal data

**Table:**

C_9_H_20_NO^+^·ClO_4_^–^	*F*_000_ = 552
*M**_r_* = 257.71	*D*_x_ = 1.376 Mg m^−^^3^
Monoclinic, *P*2_1_/*n*	Mo *K*α radiation λ = 0.71073 Å
Hall symbol: -P 2yn	Cell parameters from 2824 reflections
*a* = 7.5712 (15) Å	θ = 2.3–28.1º
*b* = 13.927 (3) Å	µ = 0.31 mm^−^^1^
*c* = 12.007 (2) Å	*T* = 113 (2) K
β = 100.71 (3)º	Block, colorless
*V* = 1244.0 (4) Å^3^	0.12 × 0.04 × 0.04 mm
*Z* = 4	

### Data collection

**Table:**

Rigaku Saturn diffractometer	2183 independent reflections
Radiation source: rotating anode	1797 reflections with *I* > 2σ(*I*)
Monochromator: confocal	*R*_int_ = 0.046
Detector resolution: 7.31 pixels mm^-1^	θ_max_ = 25.0º
*T* = 113(2) K	θ_min_ = 2.3º
ω and φ scans	*h* = −9→7
Absorption correction: multi-scan(CrystalClear; Rigaku/MSC, 2005)	*k* = −11→16
*T*_min_ = 0.963, *T*_max_ = 0.988	*l* = −13→14
7480 measured reflections	

### Refinement

**Table:**

Refinement on *F*^2^	Secondary atom site location: difference Fourier map
Least-squares matrix: full	Hydrogen site location: inferred from neighbouring sites
*R*[*F*^2^ > 2σ(*F*^2^)] = 0.045	H atoms treated by a mixture of independent and constrained refinement
*wR*(*F*^2^) = 0.127	*w* = 1/[σ^2^(*F*_o_^2^) + (0.0763*P*)^2^ + 0.0376*P*] where *P* = (*F*_o_^2^ + 2*F*_c_^2^)/3
*S* = 1.10	(Δ/σ)_max_ = 0.001
2183 reflections	Δρ_max_ = 0.60 e Å^−^^3^
157 parameters	Δρ_min_ = −0.48 e Å^−^^3^
Primary atom site location: structure-invariant direct methods	Extinction correction: none

### Special details

**Table:**

Geometry. All e.s.d.'s (except the e.s.d. in the dihedral angle between two l.s. planes) are estimated using the full covariance matrix. The cell e.s.d.'s are taken into account individually in the estimation of e.s.d.'s in distances, angles and torsion angles; correlations between e.s.d.'s in cell parameters are only used when they are defined by crystal symmetry. An approximate (isotropic) treatment of cell e.s.d.'s is used for estimating e.s.d.'s involving l.s. planes.
Refinement. Refinement of *F*^2^ against ALL reflections. The weighted *R*-factor *wR* and goodness of fit *S* are based on *F*^2^, conventional *R*-factors *R* are based on *F*, with *F* set to zero for negative *F*^2^. The threshold expression of *F*^2^ > σ(*F*^2^) is used only for calculating *R*-factors(gt) *etc*. and is not relevant to the choice of reflections for refinement. *R*-factors based on *F*^2^ are statistically about twice as large as those based on *F*, and *R*- factors based on ALL data will be even larger.

### Fractional atomic coordinates and isotropic or equivalent isotropic displacement parameters (Å^2^)

**Table:**

	*x*	*y*	*z*	*U*_iso_*/*U*_eq_	
O1	0.8859 (2)	0.66280 (10)	0.28774 (12)	0.0224 (4)	
H1	0.9165	0.6118	0.2631	0.034*	
N1	0.6419 (2)	0.74364 (13)	−0.04347 (15)	0.0172 (4)	
H1A	0.694 (4)	0.6951 (17)	−0.078 (2)	0.027 (6)*	
H1B	0.570 (4)	0.7733 (17)	−0.099 (2)	0.029 (7)*	
C1	0.7957 (3)	0.80834 (14)	0.01410 (18)	0.0200 (5)	
C2	0.8982 (3)	0.75325 (14)	0.11605 (18)	0.0216 (5)	
H2A	0.9652	0.7001	0.0882	0.026*	
H2B	0.9874	0.7969	0.1609	0.026*	
C3	0.7796 (3)	0.71199 (14)	0.19322 (16)	0.0184 (5)	
H3	0.7150	0.7661	0.2230	0.022*	
C4	0.6403 (3)	0.64456 (14)	0.12660 (17)	0.0187 (5)	
H4A	0.5646	0.6181	0.1782	0.022*	
H4B	0.7030	0.5902	0.0977	0.022*	
C5	0.5198 (3)	0.69386 (14)	0.02718 (17)	0.0188 (5)	
C6	0.3909 (3)	0.76627 (15)	0.06528 (19)	0.0240 (5)	
H6A	0.3364	0.8060	0.0006	0.036*	
H6B	0.4574	0.8073	0.1250	0.036*	
H6C	0.2963	0.7318	0.0946	0.036*	
C7	0.4117 (3)	0.62072 (15)	−0.05253 (19)	0.0248 (5)	
H7A	0.4940	0.5759	−0.0797	0.037*	
H7B	0.3397	0.6542	−0.1172	0.037*	
H7C	0.3319	0.5851	−0.0118	0.037*	
C8	0.9141 (3)	0.82429 (17)	−0.0744 (2)	0.0292 (6)	
H8A	1.0164	0.8651	−0.0422	0.044*	
H8B	0.8437	0.8557	−0.1412	0.044*	
H8C	0.9582	0.7623	−0.0965	0.044*	
C9	0.7249 (3)	0.90544 (15)	0.0458 (2)	0.0277 (5)	
H9A	0.6607	0.8970	0.1088	0.042*	
H9B	0.6427	0.9320	−0.0197	0.042*	
H9C	0.8259	0.9496	0.0685	0.042*	
Cl1	0.15810 (8)	0.46849 (3)	0.19458 (5)	0.0256 (2)	
O2	−0.0091 (3)	0.47048 (12)	0.23547 (17)	0.0431 (5)	
O3	0.2067 (3)	0.56462 (12)	0.17449 (16)	0.0423 (5)	
O4	0.1359 (3)	0.41692 (15)	0.08989 (19)	0.0627 (7)	
O5	0.2955 (3)	0.42562 (16)	0.2770 (2)	0.0603 (7)	

### Atomic displacement parameters (Å^2^)

**Table:**

	*U*^11^	*U*^22^	*U*^33^	*U*^12^	*U*^13^	*U*^23^
O1	0.0226 (9)	0.0236 (8)	0.0180 (7)	0.0044 (6)	−0.0039 (7)	−0.0023 (6)
N1	0.0133 (10)	0.0196 (9)	0.0184 (9)	0.0023 (7)	0.0026 (8)	0.0006 (7)
C1	0.0124 (11)	0.0205 (11)	0.0266 (12)	−0.0036 (8)	0.0023 (10)	−0.0006 (8)
C2	0.0135 (11)	0.0233 (11)	0.0262 (11)	−0.0014 (8)	−0.0012 (10)	−0.0017 (8)
C3	0.0153 (12)	0.0205 (10)	0.0173 (10)	0.0024 (8)	−0.0021 (9)	−0.0012 (8)
C4	0.0159 (11)	0.0203 (10)	0.0191 (11)	−0.0012 (8)	0.0009 (9)	0.0001 (8)
C5	0.0146 (12)	0.0202 (10)	0.0218 (11)	−0.0040 (8)	0.0040 (10)	0.0013 (8)
C6	0.0141 (12)	0.0292 (12)	0.0300 (12)	0.0026 (9)	0.0075 (10)	0.0034 (9)
C7	0.0222 (13)	0.0248 (11)	0.0240 (12)	−0.0065 (9)	−0.0043 (10)	0.0025 (9)
C8	0.0194 (13)	0.0350 (13)	0.0344 (13)	0.0002 (10)	0.0081 (11)	0.0099 (10)
C9	0.0241 (13)	0.0215 (12)	0.0365 (13)	−0.0024 (9)	0.0030 (11)	−0.0001 (9)
Cl1	0.0215 (4)	0.0218 (3)	0.0361 (4)	0.0047 (2)	0.0119 (3)	0.0035 (2)
O2	0.0254 (11)	0.0522 (12)	0.0575 (13)	0.0051 (8)	0.0223 (10)	0.0141 (8)
O3	0.0465 (13)	0.0299 (10)	0.0501 (11)	−0.0063 (8)	0.0079 (10)	0.0074 (8)
O4	0.0644 (16)	0.0566 (13)	0.0694 (15)	0.0081 (11)	0.0189 (13)	−0.0359 (11)
O5	0.0340 (12)	0.0705 (15)	0.0760 (15)	0.0229 (10)	0.0091 (12)	0.0437 (12)

### Geometric parameters (Å, °)

**Table:**

O1—C3	1.437 (2)	C5—C6	1.532 (3)
O1—H1	0.8199	C6—H6A	0.9800
N1—C1	1.532 (3)	C6—H6B	0.9800
N1—C5	1.532 (3)	C6—H6C	0.9800
N1—H1A	0.92 (3)	C7—H7A	0.9800
N1—H1B	0.88 (3)	C7—H7B	0.9800
C1—C9	1.528 (3)	C7—H7C	0.9800
C1—C2	1.529 (3)	C8—H8A	0.9800
C1—C8	1.529 (3)	C8—H8B	0.9800
C2—C3	1.518 (3)	C8—H8C	0.9800
C2—H2A	0.9900	C9—H9A	0.9800
C2—H2B	0.9900	C9—H9B	0.9800
C3—C4	1.523 (3)	C9—H9C	0.9800
C3—H3	1.0000	Cl1—O3	1.4210 (18)
C4—C5	1.524 (3)	Cl1—O5	1.427 (2)
C4—H4A	0.9900	Cl1—O4	1.430 (2)
C4—H4B	0.9900	Cl1—O2	1.4408 (18)
C5—C7	1.527 (3)		
			
C3—O1—H1	106.4	C4—C5—N1	107.61 (17)
C1—N1—C5	120.17 (16)	C7—C5—N1	105.26 (16)
C1—N1—H1A	106.5 (16)	C6—C5—N1	110.57 (16)
C5—N1—H1A	105.7 (15)	C5—C6—H6A	109.5
C1—N1—H1B	112.1 (16)	C5—C6—H6B	109.5
C5—N1—H1B	106.2 (16)	H6A—C6—H6B	109.5
H1A—N1—H1B	105 (2)	C5—C6—H6C	109.5
C9—C1—C2	113.22 (18)	H6A—C6—H6C	109.5
C9—C1—C8	108.82 (17)	H6B—C6—H6C	109.5
C2—C1—C8	110.68 (18)	C5—C7—H7A	109.5
C9—C1—N1	111.14 (18)	C5—C7—H7B	109.5
C2—C1—N1	107.24 (16)	H7A—C7—H7B	109.5
C8—C1—N1	105.46 (17)	C5—C7—H7C	109.5
C3—C2—C1	114.12 (18)	H7A—C7—H7C	109.5
C3—C2—H2A	108.7	H7B—C7—H7C	109.5
C1—C2—H2A	108.7	C1—C8—H8A	109.5
C3—C2—H2B	108.7	C1—C8—H8B	109.5
C1—C2—H2B	108.7	H8A—C8—H8B	109.5
H2A—C2—H2B	107.6	C1—C8—H8C	109.5
O1—C3—C2	110.75 (17)	H8A—C8—H8C	109.5
O1—C3—C4	110.62 (16)	H8B—C8—H8C	109.5
C2—C3—C4	110.06 (16)	C1—C9—H9A	109.5
O1—C3—H3	108.4	C1—C9—H9B	109.5
C2—C3—H3	108.4	H9A—C9—H9B	109.5
C4—C3—H3	108.4	C1—C9—H9C	109.5
C3—C4—C5	112.87 (16)	H9A—C9—H9C	109.5
C3—C4—H4A	109.0	H9B—C9—H9C	109.5
C5—C4—H4A	109.0	O3—Cl1—O5	109.47 (13)
C3—C4—H4B	109.0	O3—Cl1—O4	108.40 (13)
C5—C4—H4B	109.0	O5—Cl1—O4	110.53 (14)
H4A—C4—H4B	107.8	O3—Cl1—O2	108.16 (11)
C4—C5—C7	111.31 (16)	O5—Cl1—O2	110.23 (11)
C4—C5—C6	112.63 (16)	O4—Cl1—O2	110.00 (13)
C7—C5—C6	109.21 (18)		
			
C5—N1—C1—C9	−76.1 (2)	O1—C3—C4—C5	178.28 (15)
C5—N1—C1—C2	48.2 (2)	C2—C3—C4—C5	−59.0 (2)
C5—N1—C1—C8	166.19 (18)	C3—C4—C5—C7	167.26 (17)
C9—C1—C2—C3	72.5 (2)	C3—C4—C5—C6	−69.7 (2)
C8—C1—C2—C3	−165.05 (17)	C3—C4—C5—N1	52.4 (2)
N1—C1—C2—C3	−50.5 (2)	C1—N1—C5—C4	−49.6 (2)
C1—C2—C3—O1	−179.04 (15)	C1—N1—C5—C7	−168.37 (17)
C1—C2—C3—C4	58.3 (2)	C1—N1—C5—C6	73.8 (2)

### Hydrogen-bond geometry (Å, °)

**Table:**

*D*—H···*A*	*D*—H	H···*A*	*D*···*A*	*D*—H···*A*
N1—H1A···O4^i^	0.92 (3)	2.05 (3)	2.914 (3)	157 (2)
N1—H1B···O1^ii^	0.88 (3)	1.97 (3)	2.847 (3)	173 (2)
O1—H1···O2^iii^	0.82	2.09	2.896 (2)	167
O1—H1···Cl1^iii^	0.82	2.93	3.6985 (16)	158

Symmetry codes: (i) −*x*+1, −*y*+1, −*z*; (ii) *x*−1/2, −*y*+3/2, *z*−1/2; (iii) *x*+1, *y*, *z*.
